# Loss of control as a violation of expectations: Testing the predictions of a common inconsistency compensation approach in an inclusionary cyberball game

**DOI:** 10.1371/journal.pone.0221817

**Published:** 2019-09-09

**Authors:** Michael Niedeggen, Rudolf Kerschreiter, Katharina Schuck

**Affiliations:** 1 Division of Experimental Psychology and Neuropsychology, Department of Education and Psychology, Freie Universität Berlin, Berlin, Germany; 2 Division of Social, Organizational, and Economic Psychology, Department of Education and Psychology, Freie Universität Berlin, Berlin, Germany; Temple University, UNITED STATES

## Abstract

Personal control relies on the expectation that events are contingent upon one’s own behavior. A common ‘*inconsistency compensation approach*’ posits that a violation of expectancies in social interaction triggers aversive arousal and compensatory effort. Following this approach, we tested the hypothesis that interventions affecting participants' decisions violate the expected personal control. In a modified version of the established cyberball paradigm, participants were not excluded, but consistently included. However, their decisions regarding the recipient of a ball throw in the virtual game were occasionally overruled (*expectancy violation*). We hypothesized that this intervention will trigger a P3 response in event-related brain potentials (ERP). Since this component is related to subjective expectancies, its amplitude was assumed to depend on the frequency of interventions (independent factor: loss of control). Further, we manipulated the vertical position of the participants’ avatar on the computer screen (independent factor: verticality). Building on research showing that verticality is related to the self-assigned power and influences the expected level of control, we hypothesized that the ERP effects of intervention should be more pronounced for participants with avatars in superior position. As predicted, both experimental factors interactively affected the expression of the ERP response: In case of low intervention frequency, P3 amplitudes were significantly pronounced if the participants’ avatar was positioned above as compared to below co-players (high > low self-assigned power). The effect of verticality could be traced back to a lack of adaptation of P3 amplitudes to recurring aversive events. By demonstrating that loss of control triggers ERP effects corresponding to those triggered by social exclusion, this study provides further evidence for a common cognitive mechanism in reactions to aversive events based on an inconsistency in expectancy states.

## Introduction

People want control in their lives. Personal control is a central human need that relies on the expectation that events are contingent upon one’s own behavior (Rotter, 1966). It embraces the concept of *choice*, the ability to select options [1]. A loss of control is reported to be stressful and anxiety provoking [2–4] and elicits the activation of a compensatory control mechanism [5].

The current study examined whether a loss of control defines an inconsistency in human information processing which relies on a violation of subjective expectancies. Based on previous experiments on social exclusion [6, 7], we supposed that the frequency of an aversive intervention as well as the self-assigned social power will affect experienced inconsistency. In the current research, event-related brain potentials (ERPs) allowed us to track the dynamics of the participants’ state of expectancy and to explore differences in adaptation to recurring aversive events.

### Inconsistency compensation approach

Both, a loss of control [1, 8] and social exclusion [9, 10] [11, 12], induce a threat of our social needs and consequently an immediate aversive experience which directly impacts our affective state [3]. For both aversive states, psychological models have been proposed and tested in numerous behavioral, psychophysiological, and neuroimaging studies (for control, see: [13, 14] for exclusion: [15, 16]).

In contrast to these specific models, an overarching ‘inconsistency compensation’ approach [17] provides a more general framework and therefore meets the recent call for more general theories on human behavior [18]. Following the tradition of ‘cognitive dissonance theory’ [19, 20], inconsistencies are triggered by the violation of subjective expectations, beliefs, or goals. These violations will evoke an aversive arousal and, consequently, a compensatory effort, such as accommodation, assimilation, or affirmation [21]. Neuroimaging results support the notion that the detection of inconsistencies relies on the activation of common neural structures [22, 23]. Following this unified motivational account, most social psychological phenomena [17], including social exclusion and loss on control, therefore rely on the violation of subjective expectations.

In the following, we will first show how the predictions of this *expectancy violation account* have been examined in the context of social exclusion. We will introduce the experimental paradigm (cyberball) and the electrophysiological marker of an expectancy violation, the P3 component. After that, we will demonstrate how this approach can be applied in the context of a loss of control.

### Expectancy violation and social exclusion

In the case of social exclusion, the predictions of an approach based on expectancy violation have already been studied intensively. Experimental approaches mostly rely on the cyberball paradigm [24]. Here, the participant is exposed to exclusion by two (putative) co-players in a computerized ball tossing game. The short-lived experience of exclusion induces a reliable threat of fundamental human needs, namely belonging and self-esteem [25], and appears to provide a valid simulation of a real-life experience.

In neuroimaging studies, neural structures associated with the processing of subjective expectancies were elicited by social rejection [26]. Electrophysiological studies identified markers for a violation of expectancies in EEG activity [27] as well as in event-related brain potentials (ERPs). Among the several components discussed in previous studies [28, 29], the most-promising candidate is the well-known P3 wave, a late positive deflection at 300–500 ms. The P3 can be elicited in numerous cognitive paradigms and can be related to different stages in attentive and mnestic processing [30, 31]. The cyberball paradigm shares the characteristics of the oddball paradigm [32] which probes the brain’s response to casual relevant target events. Here, the P3 amplitude is inversely related to the *subjective* probability of the target relevant event [33, 34]. In the exclusionary cyberball, the ball reception can be defined as a target event. Corresponding to the findings in the oddball paradigm, an increase in P3 amplitude can be reliably elicited if the probability of ball receptions is reduced (i.e. from 33% to 16% in a setup with two co-players, see [35]). Most importantly, this P3 effect is not a mere reflection of event probability, but critically relies on the participants’ subjective expectancies: If the reduced involvement is expected (by increasing the number of co-players, see [36]), the P3 amplitude is not affected, and the reduction in ball reception is consequently not rated as aversive in the post-hoc questionnaires. This pattern of results demonstrates that the P3 effect depends on the violation of expected involvement.

As shown in a previous P3 study focusing on the effect of stereotyped cues in a Lunchroom task, the level of expected involvement can differ between groups of participants [37]. This finding corresponds to clinical studies with borderline patients showing that the expression of the P3 effect and the self- reports depend on the *a priori* level of expected social participation [38, 39]. A corresponding bias can also be induced experimentally in healthy participants by manipulating the self-assigned social power. In several experimental studies, self-assigned social power was effectively manipulated by the assigned vertical position [40] which leads to a sense of entitlement [41]. This effect of *verticality* has been transferred to the cyberball paradigm: In ERP studies [6, 42], the participants’ avatar was either positioned above (*superior*) or below (*inferior*) the avatars of the co-players. In participants with an avatar at a superior position, the P3 effect was more expressed in an exclusionary condition. Ruling out that the ERP effect was due to a perceptual or attentive bias in the processing of the upper visual field [6], the P3 effect indicates that participants assigned to a superior position as compared to participants assigned to an inferior position are less prepared for exclusionary events. Correspondingly, the superior group also rated exclusion as more-aversive in a retrospective questionnaire. In sum, the pattern of results supports the notion that self-assigned social power heightens the sensitivity for social exclusion [43].

According to recent ERP findings [42], this verticality effect is associated with a differential adaptation to the aversive event: Whereas the P3 effect is gradually reduced within an experimental run in participants with an avatar at inferior position, it remains stable in participants with an avatar at superior position.

In sum, these ERP findings revealed that the processing of social exclusion is congruent with the predictions of an *expectancy violation* (and the overarching *inconsistency compensation*) approach: The P3 does not only reflect the violation of expected participation, but also the bias in the level of expectancy (induced by verticality). In the following, we will demonstrate how this approach can be applied to the processing of a loss of control.

### The present study: Expectancy violation and loss of control

Provided that the predictions of an expectancy violation approach do not exclusively apply to the processing of exclusion, we expect that a loss of control will trigger a comparable ERP signature. To test this, in the present study we modified the cyberball setup to focus on the effect of loss of personal control. In the *exclusionary* cyberball, participation is reduced but decisional autonomy is provided: The participant is free to select the recipient of her/his ball throw. In the modified *intervention* cyberball we introduce in this research, the participant is included in the game, but personal control is challenged by a putative supervisor who can overrule the participants’ decision and select a different recipient of the participants' ball throw. In contrast to the established *exclusionary* cyberball, the modified *intervention* cyberball therefore controls for belonging (inclusion of the participant) and aims to selectively threaten the need for control (intervention).

The experimental setup allows us to test the predictions of an expectancy violation account with respect to the loss of control. Two factors affecting the violation of expectancies were manipulated, intervention frequency and vertical position of the participants’ avatar on the screen. Furthermore, the ERPs allow us to monitor a covert process: the adaptation to the recurring intervention events. Based on previous ERP results in the exclusionary cyberball, we hypothesized the following:

#### Hypothesis 1: An intervention in the participants' decisional autonomy in the modified cyberball game elicits a P3

In prior studies running the *exclusionary* cyberball, analysis was focused on the relevant target event, the participant’s ball reception. Comparable to the oddball paradigm [33], this target event elicited a centro-parietal P3b component, with its amplitude depending on event probability [36]. The centro-parietal component has been related to stimulus evaluation in the context of preceding information (context updating [30]). In the *intervention* cyberball, analysis is focused on a deviant event (non-intended recipient of ball throw) not related to an immediate response by the participant. Following previous ERP research, a deviant is supposed to trigger an additional earlier fronto-central P3a component [31] which is related to the activation of a frontal attention network [44], but also to the certainty on upcoming events [45]. We therefore assumed that the P3 amplitudes will reflect the predictability of the deviant event as defined by the probability of intervention.

#### Hypothesis 2: The expression of the P3 induced by an intervention in the participants' decisional autonomy is more pronounced in participants with an avatar at superior position as compared to an inferior position

As mentioned above, the expectation for participation can be biased by vertical position [6, 42]. Following a more-general ‘inconsistency compensation’ approach [17], the effect of verticality on the self-assignment of social power [40] should also apply for self-assigned personal power [46]. Accordingly, we assumed that the expression of the P3 responses to intervention should be more pronounced in participants with an avatar at superior position. In line with previous ERP findings from exclusionary cyberball [6] we expected this effect to be more pronounced if the predictability of an intervention is low.

#### Hypothesis 3: The differences in the expression of the P3 induced by verticality can be traced back to differential adaptation effects

ERP studies are based on averaging the response to repeatedly presented events. Consequently, relevant events (e.g. ball reception or interventions, respectively) must also occur frequently in a cyberball game. In the exclusionary cyberball, physiological responses to recurring relevant events (here: ball reception) change within an experimental block over time [47]. A corresponding decrease of the P3 amplitude [28] can be related to a re-adjustment of subjective expectancies [48]. Recent ERP results suggest that the aforementioned effect of verticality is due to a differential adaptation of the P3 amplitude [42]: If a participant is assigned to an inferior position, the decrease in P3 amplitude is markedly expressed. In contrast, P3 amplitudes remain constant if a superior position has been assigned. We suppose that the same process can be observed in the intervention cyberball game. In other words, in line with evidence from exclusionary cyberball studies we hypothesized that the P3 will also reflect an adjustment of expected interventions and that this effect will depend on the assigned vertical position of the participant’s avatar.

## Materials and methods

The experimental procedure was approved by the local ethics committee at the FU Berlin (No.006.2019). All participants provided written consent for participation according to the Declaration of Helsinki. The experiment reported in this article was not formally preregistered. Pre-processed data used for statistical testing are available online. Requests for the source code of the experimental procedure can be sent via email to the lead author. We report all measures, manipulations and exclusions.

### Participants

Sample size was determined a priori using G*Power [49]. Previous ERP studies reported large effects of the within-participant experimental factor ‘probability for ball reception’ (here: frequency of intervention), and medium effects of the between-participant experimental factor ‘verticality’ [6]. Our power analysis was set out to replicate the crucial interaction of the experimental factors. To detect a medium effect of the within- and the between-factor (*f* = 0.20 adjusted to the taxonomy of Cohen) with a power of 80% using an *F*-test with alpha at .05 a sample size of 52 participants was required.

The required number of participants (n = 52, 36 female, 16 male, age range: 18 to 36 years) was included in the final analysis. Data of 14 additional participants (10 female, 4 male) were recorded, but rejected following a rigorous artifact correction (criteria: see below). Excluded participants did not differ from the included with respect to age (*t*(64) = -.812, *p* = .42). Participants were randomly assigned to the conditions of the factor ‘verticality’: Each of the experimental groups comprised 26 participants (‘superior‘: 16 female, 10 male, age: M = 25.96, SD = 5.89; ‘inferior‘: 20 female, 6 male, age: M = 22.50, SD = 3.15). Please note that the difference in age between the groups was statistically significant (*t*(50) = 2.64, *p* = .011), and will be considered in the statistical analysis.

### Task and design

The experimental setup (programmed in PsychoPy, v1.8, [50]) was a modification of the ERP-adjusted cyberball game previously reported [36]. The cover story of participating in a visual imagination study was supported by an initial questionnaire about the participants’ visual imagination ability (Vividness of Visual Imagery Questionnaire, [51]).

**Fig 1:**
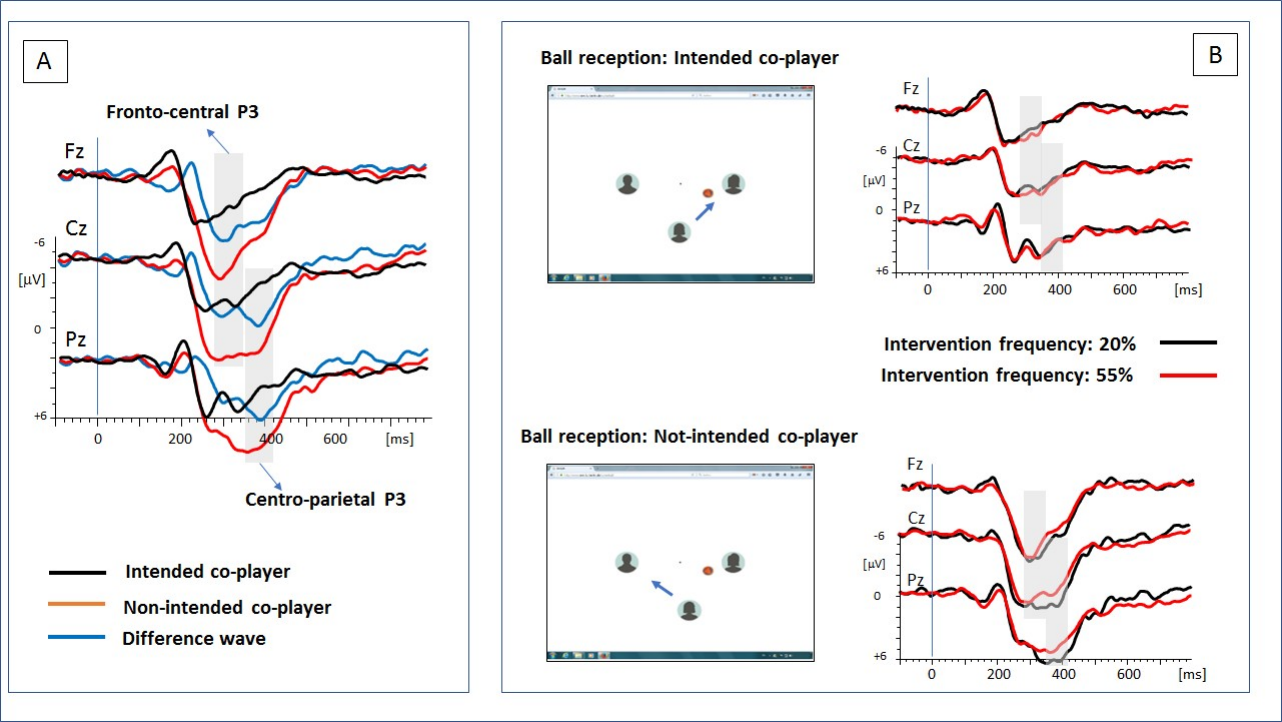
(A) Grand-averaged ERPs at midline electrodes Fz, Cz, and Pz separated for the experimental factor recipient (‘ball reception by intended co-player’ vs. ‘ball reception by non-intended co-player’). The difference waves illustrate two positive components: an early fronto-central P3a (290–350 ms) followed by a centro-parietal P3b (350–410 ms). (B) ERPs effects of the experimental factor intervention frequency (20% vs 55%) separated for the ball reception of the intended and non-intended co-player. Modulation of the P3 amplitudes—time ranges of the P3a and P3b are shaded—was restricted to the deviant event (non-intended co-player).

The setup of the following *intervention* cyberball game is depicted in Fig 1A: All players—putatively connected via internet—are represented by three avatars on the computer screen (7° x 7° at a viewing distance of 120 cm). Participants were previously asked to select an avatar of their choice [52] which was centered horizontally on the computer screen. Each participant was quasi-randomly assigned (experimental factor: verticality) to a vertical position below (*inferior*) or above (*superior*) of the co-players avatars. The vertical position of the avatars of the two putative co-players was centered. Spatial distance between the avatars was held constant (3°) in each of the experimental conditions.

Presentation of the ball in spatial proximity to the participants' avatar signaled ball possession and requested its forwarding to one of the co-players by pressing a corresponding button on a keyboard. After this decision, the ball vanished for 500 ms before appearing in spatial proximity to a co-player’s avatar. Ball possession of the co-player lasted randomly between 400 and 1.400 ms to simulate the temporal variability of the decisional process. In each of the two experimental blocks–comprising 250 trials each—participants received the ball with a probability of 33% providing a constant inclusionary setting.

Before each experimental block, a picture of a meadow or a beach was shown accompanied by an instruction to visualize a ball throwing game at this place. Furthermore, participants were informed that a ‘supervisor’—who is not a co-player—might intervene in the decision of the players and that the supervisor’s activity will vary randomly between the two blocks. In case of an intervention, the ball was passed to the *non-intended* instead of the *intended* recipient. Frequency of intervention (within-participant factor: intervention) was at 20% in the first, and at 55% in the second block. Since the participant receives the ball in 83 trials in each block, the corresponding number of interventions was 17 in the first and 46 in the second block. The order of conditions was held constant in this study since previous ERP studies using the *exclusionary* cyberball provided evidence for contrast effects ([35], see discussion). Results of behavioral pilot studies indicated that this experimental setup selectively affected participants' need for control.

Immediately following the completion of the second block of the cyberball game, participants were asked to fill out two questionnaires referring each to the two preceding experimental blocks (low followed by high interference). The questionnaires included an estimation of the intervention frequency, the NTQ (Need Threat Questionnaire (NTQ), [24, 53]) and a rating of the self-assigned personal and social power. The analysis will focus on four scales, (1) the estimated frequency of intervention, (2) the threat of control (NTQ), (3) the self-assigned social power (two items, i.e. *I felt in charge of others*), and (4) the self-assigned personal power (two items, i.e. *I felt independent*). The power items [54] were to be estimated on a 5-point scale ranging from *none* (1) to *very much* (5).

Pilot studies indicated that an increase of intervention frequency from 20% to 55% induces a selective threat to the need for control (NTQ scale: *F*(1,40) = 4.69, *p* = .036, η_p_^2^ = .105). Other NTQ scales were not affected. The following description of results will also focus on the scale ‘control’, but data of additional NTQ scales are available as supplementary data (Tables D and E in S1 Appendix). Please note, that no significant effects were observed on the scales “belonging”, “self-esteem”, and “meaningful existence”. After completing the questionnaires, participants were fully debriefed and gave informed consent.

Since a speeded response was not required, response times were not recorded in this study. A previous experiment on social exclusion provided no evidence that the experimental factors (frequency of the aversive event, verticality) will affect the participants’ response time [7].

### EEG recording

EEG data were recorded from five active electrode positions (Fz, Cz, Pz, P7, P8) using Ag/AgCl electrodes embedded in an elastic cap (EASYCAP, Herrsching, Germany; BrainAmps amplifier, BrainProducts, Gilching, Germany). Signals from active EEG electrodes (impedance < 5 kOhm) were referenced to linked earlobes. Electrode position FCz served as ground. Ocular artefacts were controlled for by recording vertical and horizontal electrooculogram (EOG). EEG data were recorded continuously (sample rate: 500 Hz), and band-pass filtered online (0.1–100 Hz).

Off-line, EEG data were analyzed running ‘Vision Analyzer’ (Version: 2.1, Brain Products, Gilching, Germany). EEG was epoched according to the onset of ball reception of the player, the *intended* and *non-intended* co-player. Each single (epoch length: -100 to 800 ms) was filtered (0.3 to 30 Hz, 12 dB/Oct), and baseline-corrected (-100 to 0 ms). Trials were automatically excluded from analysis if they contained ocular artifacts (EOG > 50 μV). Trials were marked if an amplitude criterion was exceeded (EEG > 80 μV). In a subsequent manual correction, marked trials were inspected for EEG alpha activity, slow linear drifts, or high frequency bursts. In the first block, the probability of the events of interest (*recipient*: intended vs. non-intended) was not balanced. Therefore, the number of EEG segments with "intended" ball recipients was adjusted to the number of segments with "non-intended" ball recipients by random selection in each participant.

In the crucial experimental condition (ball reception by non-intended player), ERPs relied on a mean of 16.09 trials (SD 1.98, range 14–21 trials) in the first block, and on a mean of 27.40 trials (SD 6.58, range 16–43 trials) in the second block. The number of artefact-free trials did not differ between the experimental groups (inferior vs. superior), neither in the first (mean trial number: 16.34 vs. 15.84, *t*(50) = .908, *p* = .368) nor in the second (mean trial number: 27.81 vs. 27.00, *t*(50) = .439, *p* = .662) block.

### Data analysis

*Questionnaire data*: The three scales of interest (estimated frequency of intervention, social and personal power) were analyzed separately running 2 x 2 ANOVAs, including the between-participant factor *verticality* and the within-participant factor *frequency of intervention* (SPSS version 22, IBM). Reported degrees of freedom and p-values were corrected according to Greenhouse-Geisser. In case of a significant interaction, post-hoc comparisons were performed.

To account for the difference in mean age with respect to the between-participant factor *verticality*, all statistical effects including this factor were additionally corrected: To this end, an ANCOVA was computed including the covariate *age of participant*. All ANCOVA results reported in the following section are indexed by an asterisk (*).

*ERP data*: In a first step of analysis, ERPs of each participant were separately averaged for the different *recipient* outcomes (*intended* vs *non-intended* recipient), the experimental factor *frequency of intervention* (low vs high), and the electrode positions. If an ERP average relied on less than 15 trials, the participants’ data were discarded from analysis (rejection of 14 participants).

Following the inspection of the grand-averaged ERP difference waves (Δ[recipient non-intended–recipient intended], see Fig 1B), we identified the expected fronto-central P3a and centro-parietal P3b. Based on the peaks of the differences waves, we assigned two temporal windows of each 60 ms to the P3a (290–350 ms) and the subsequent P3b (350–410 ms). For each participant, mean amplitudes in these time windows were computed for the ERP data separated for the experimental conditions and electrodes. Since maximum peaks within each time window could not be identified reliably in single participants, peaks and latencies were not analyzed.

An ANOVA (non-intended outcome: electrode position x temporal window) confirmed that distribution of mean amplitudes at midline electrodes was significantly different in the P3a and P3b segment (electrode position x temporal windows: *F*(1,50) = 42.83, *p* < .001, η_p_^2^ = .452). As indicated by these topographical differences (see Fig 1B), electrodes Fz and Cz were summarized in the analysis of the early P3 component, respectively Cz and Pz in the analysis of the late P3. This combination has already been used in previous ERP studies on social exclusion [35]. Exported amplitude data of both P3 components were analyzed separately running 2 x 2 x 2 ANOVAs, including the between-participant factor *verticality*, and the within-participant factors *frequency of intervention* and *recipient* (SPSS version 22, IBM). The ANOVA results are reported with Greenhouse-Geisser corrected degrees of freedom and p-values. Post-hoc comparisons were motivated by significant interactions of the experimental factors.

As mentioned above, mean age of the participants differed between the experimental groups: Accordingly, all effects including the between-participant factor *verticality* were additionally corrected by running an ANCOVA including the covariate *age of participant*.

In a second step of data analysis, ERP data were separately averaged within the first and second half of each experimental block. In case of high frequent deviants (here: block 2), a rapid adaptation to the recurring signal is highly likely. Therefore, the analysis was focused on the averaged response to the first ten deviant in the first, and the averaged response to the final ten deviants in the second half. Averaged data were based on the artifact-free set of preprocessed single trials and included at least seven single trials. For each half, the mean amplitudes in the time range of the early and late P3 component were computed. The statistical analysis was focused on the event ‘non-intended recipient’ and comprised the within-participant factors *half* (first vs. second), *frequency of intervention* (low vs. high), and the between-participant factor *verticality* (inferior vs superior). If the corresponding 2 x 2 x 2 ANOVA indicated a significant interaction, post-hoc comparisons were performed. Effects of the factor *verticality* were additionally controlled for the effect of the covariate *age of participant*.

Although the number of averaged trials used in the in the split-half analyses is quite low (block 1: 8.05, block 2: 13.70), we assume that the reliability of the ERP signal is provided: For each participant, we computed the correlation coefficient between the averaged ERP signal of the first and second half. As for the crucial first block (low intervention frequency), the mean correlation coefficient was .67 (SD .14) at electrode position Cz—indicating a high reliability.

Moreover, it is important to note that the results of the split-half analysis (see below) replicates the results of an earlier ERP study on social exclusion [7]. Finally, the P3 amplitude is a prominent ERP component which has previously been used in single-trial analysis [48].

ERPs evoked by the event ‘ball reception of the participant’ (*self*) will not be considered in the result section: Neither the amplitude in the range of the P3a nor in the range of the P3b was affected by the experimental factor *verticality* (P3a: *F*(1,50) = 0.01, *p* = .924, η_p_^2^ = .000, P3b: *F*(1,50) = .086, *p* = .357, η_p_^2^ = .017). This pattern also applies for the interaction of the experimental factors *intervention frequency* and *verticality* (P3a: *F*(1,50) = 0.08, *p* = .777, η_p_^2^ = .002, P3b: *F*(1,50) = .009, *p* = .768, η_p_^2^ = .002). Details can be found in the supplementary data (Figure A and B in S1 Appendix, Tables A to C in S1 Appendix).

## Results

The descriptive questionnaire data and the corresponding results of the inference statistics are presented in Tables 1 and 2. Statistical results in the following sections always refer to post-hoc comparisons indicated by significant interactions.

**Table 1:** Descriptive statistics and ANOVA results for questionnaire data, separated for intervention frequency (IF, 20% vs. 55%) and group assignment (vertical position: inferior vs. superior). To each mean value, upper and lower limits of confidence intervals (95%) are additionally provided. ANOVA results for the factor vertical position are additionally controlled for the covariate 'age of participant' in an ANCOVA. All ANCOVA results are indexed by an asterisk (*).

Intervention Frequency	Intervention Frequency (IF) 20%		Intervention Frequency (IF) 55%	
Vertical Position	Inferior	Superior	Inferior	Superior
**Estimated Intervention Frequency**	24.43% (18.77%, 30.08%)	30.12% (24.35%, 35.88%)	43.96% (36.02%, 51.91%)	37.16% (29.36%, 45.26%)
Effect (IF)	*F(1*,*50) = 29*.*47*, *p <* .*001*, *η*_*p*_^*2*^ *= 0*.*371*			
Effect (Position)	F(1,50) = .015, p = .904, η_p_^2^ = .000 (*F(1,49) = .22, p = .640, η_p_^2^ = .004)			
Effect (IF x Position)	*F(1*,*50) = 5*.*27*, *p =* .*026*, *η*_*p*_^*2*^ *= 0*.095 (*F(1,*49) = 4*.*44*, *p =* .*040*, *η*_*p*_^*2*^ *=* .*083*)			
**NTQ scale: Control**	2.35 (2.04, 2.66)	2.28 (1.97, 2.59	2.09 (1.81, 2.37)	2.04 (1.76, 2.32)
Effect (IF)	*F(1*,*50) = 6*.*09*, *p =* .*017*, *η*_*p*_^*2*^ *=* .*108*			
Effect (Position)	F(1,50) = .009, p = .763, η_p_^2^ = .002 (*F(1,49) = .18, p = .675, η_p_^2^ = .004)			
Effect (IF x Position)	F(1,50) = 0.08, p = .930, η_p_^2^ = .000 (*F(1,49) = .07, p = .790, η_p_^2^ = .001)			
**Rating of self-assigned social power**	2.54 (2.18, 2.83)	2.62 (2.26, 2.98)	2.32 (1.94, 2.69)	2.72 (2.35, 3.09)
Effect (IF)	F(1,50) = 0.11, p = .738, η_p_^2^ = .002			
Effect (Position)	F(1,50) = .94, p = .336, η_p_^2^ = .019 (*F(1,49) = 1.35, p = .251, η_p_^2^ = .027)			
Effect (IF x Position)	F(1,50) = 1.19, p = .280, η_p_^2^ = .022 (*F(1,49) = 1.10, p = .299, η_p_^2^ = .022			
**Rating of self-assigned personal powe**r	3.00 (2.60, 3.39)	3.25 (2.85, 3.64)	2.96 (2.52, 3.41)	2.69 (2.26, 3.13)
Effect (IF)	*F(1*,*50) = 5*.*29*, *p =* .*026*, *η*_*p*_^*2*^ *=* .*096*			
Effect (Position)	F(1,50) = .012, p = .914, η_p_^2^ = .000 (*F(1,49) = .222, p = .643 ., η_p_^2^ = .004)			
Effect (IF x Position)	*F(1*,*50) = 5*.*30*, *p =* .*026*, *η*_*p*_^*2*^ *= 0*.096 (*F(1,*49) = 3*.*58*, *p =* .*064*, *η*_*p*_^*2*^ *=* .*068)*			

**Table 2:** Descriptive statistics and ANOVA results for the ERP data, separated for intervention frequency (IF, 20% vs. 55%), group assignment (vertical position: inferior vs. superior), and outcome of ball though (RC, intended vs. non-intended recipient). To each mean value, the upper and lower limits of confidence intervals (95%) are additionally provided. ANOVA results for the factor vertical position are additionally controlled for the covariate 'age of participant' in an ANCOVA. All ANCOVA results are indexed by an asterisk (*).

Intervention Frequency	Intervention Frequency (IF) 20%		Intervention Frequency (IF) 55%	
Vertical Position	Inferior	Superior	Inferior	Superior
**P3a amplitude (290–350 ms), *intended outcome (RC)***	2.47 μV (1.41 μV, 3.55 μV)	2.58 μV (1.52 μV, 3.65 μV)	2.36 μV (1.29 μV, 3.33 μV)	3.28 μV (2.32 μV, 4.26 μV)
**P3a amplitude (290–350 ms), *non-intended outcome (RC)***	5.73 μV (4.78 μV, 7.30 μV)	8.38 μV (6.84 μV, 9.92 μV)	5.31 μV (4.06 μV, 6.58 μV)	6.04 μV (4.19 μV, 7.27 μV)
Effect (RC)	*F(1*,*50) = 112*.*32*, *p <* .*001*, *η*_*p*_^*2*^ *=* .*692*			
Effect (IF)	F(1,50) = 3.02, p = .088, η_p_^2^ = .057			
Effect (Position)	F(1,50) = 2.67, p = .108, η_p_^2^ = .051 (*F(1,49) = 2.54, p = .117, η_p_^2^ = .049)			
Effect (RC x IF)	*F(1*,*50) = 9*.*32*, *p =* .*004*, *η*_*p*_^*2*^ *=* .*157*			
Effect (RC x Positon)	F(1,50) = 2.83, p = .099, η_p_^2^ = .054 (*F(1,49) = 3.65, p = .062, η_p_^2^ = .069)			
Effect (IF x Position)	F(1,50) = 0.79, p = .378, η_p_^2^ = .016 (*F(1,49) = 0.65, p = .424, η_p_^2^ = .013)			
Effect (RC x IF x Position)	*F(1*,*50) = 6*.*34*, *p =* .*015*, *η*_*p*_^*2*^ *=* .113 (*F(1,*49) = 8*.*42*, *p =* .*006*, *η*_*p*_^*2*^ *=* .*147*)			
**P3b amplitude (235–410 ms), *intended outcome (RC)***	2.50 μV (1.41 μV, 3.60 μV)	2.39 μV (1.30 μV, 3.48 μV)	2.40 μV (1.19 μV, 3.62 μV)	2.13 μV (0.92 μV, 3.35 μV)
**P3b amplitude (350–410 ms), *non-intended outcome (RC)***	6.46 μV (4.87 μV, 8.06 μV)	7.28 μV (5.68 μV, 8.87 μV)	5.18 μμV (3.78 μV, 6.5 μV)	5.44 μV (4.05 μV, 6.82 μV)
Effect (RC)	*F(1*,*50) = 105*.*33*, *p <* .*001*, *η*_*p*_^*2*^ *=* .*678*			
Effect (IF)	*F(1*,*50) = 6*.*71*, *p =* .*013*, *η*_*p*_^*2*^ *=* .*118*			
Effect (Position)	F(1,50) = 0.05, p = .816, η_p_^*2*^ = .001 (*F(1,49) = 0.49, p = .486, η_p_^*2*^ = .010)			
Effect (RC x IF)	*F(1*,*50) = 4*.*44*, *p =* .*040*, *η*_*p*_^*2*^ *=* .*082*			
Effect (RC x Positon)	F(1,50) = 1.00, p = .322, η_p_^*2*^ = .020 (*F(1,49) = 0.98, p = .328, η_p_^*2*^ = .020)			
Effect (IF x Position)	F(1,50) = 0.28, p = .599, η_p_^*2*^ = .006 (*F(1,49) = 0.21, p = .651, η_p_^*2*^ = .004)			
Effect (RC x IF x Position)	F(1,50) = 0.09, p = .759, η_p_^*2*^ = .002 (*F(1,49) = 0.624, p = .434, η_p_^*2*^ = .013)			

### Manipulation check and ratings

**Fig 2:**
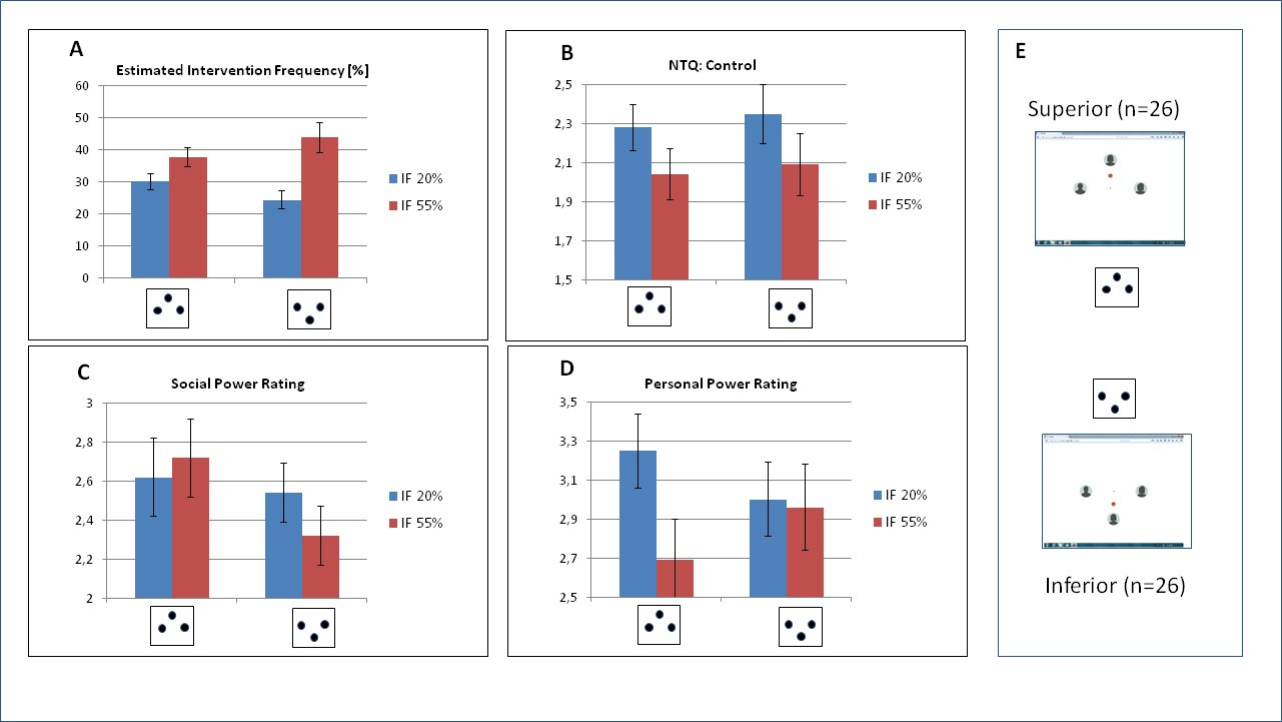
Descriptive statistics for the self-report data, separated for experimental factors ‘position’ (inferior vs. superior) and ‘intervention frequency’ (IF 20% vs IF 55%). Error bars refer to the standard error of mean. (A) Estimated frequency of intervention was overestimated by participants in superior position in the first block. (B) The threat to the need for control was increased by increasing intervention frequency. (C) Ratings of self-assigned social power were not affected by experimental manipulation. (D) In contrast, ratings of self-assigned personal power were reduced by increasing the intervention frequency, but exclusively in the superior group. (E) Explanation of the pictograms used.

The participants noticed the increasing frequency of the supervisors’ activity reliably. Although the level of estimated frequency did not differ between the two *intervention* groups, a significant interaction of the experimental factors was indicated (Table 1, Fig 2A). As compared to the ‘inferior’ group, *F*(1,25) = 19.47, *p* < .001, η_p_^*2*^ = .438, effect size for the factor *intervention frequency* was markedly reduced in the ‘superior’ group, *F*(1,25) = 10.49, *p* = .003, η_p_^*2*^ = .296. In the latter group, intervention frequency was overestimated in the first, and underestimated it in the second block.

In accordance with our pilot studies, the only NTQ scale affected by experimental manipulation was ‘control’ (Table 1, Fig 2B). The need for control was significantly threatened by increasing the frequency of intervention.

In the intervention cyberball, ratings of self-assigned social power (i.e. *I felt in charge of others*) were neither affected by the increasing frequency of intervention, nor by the vertical position (Table 1, Fig 2C). In contrast, rating of self-assigned personal power (*I felt independent*), was rated significantly lower if intervention frequency was increased, and vertical position moderated this effect significantly (Table 1, Fig 2D). If participants were assigned to an avatar at superior position, the rating of self-assigned personal power was more expressed in the first block but dropped markedly in the second block. Post-hoc comparisons confirmed that the effect of *intervention frequency* was exclusively expressed in the ‘superior’, *F*(1,25) = 10.83, *p* = .003, η_p_^*2*^ = .302, but not in the ‘inferior’ group, *F*(1,25) = .00, *p* = .999, η_p_^*2*^ = .000. Please note that the crucial interaction between vertical position and intervention frequency is on the verge of significance when the covariate ‘age of participant’ is considered. This will be further considered in the discussion.

### ERP data: Analysis between experimental blocks

As mentioned above, further reports will focus on the different outcomes of a participants’ ball toss, the reception of the intended vs. the reception of the non-intended co-player. Analysis of the event ‘self’ (participant receives the ball) can be found in the supplementary material (Figure A and B in S1 Appendix).

As depicted in Fig 1A, the grand-averaged ERPs were characterized by a sustained positivity starting at about 250 ms. The positivity is clearly more expressed for the event ‘recipient non-intended’ when compared to the event ‘recipient intended’. Based on the ERP difference wave, two positive components were identified representing the early fronto-central P3a peaking at about 310 ms, followed by a late centro-parietal P3b peaking at about 400ms. Analyses were based on the mean amplitudes in two temporal ranges (mean[Fz, Cz]: 290–350 ms, mean[Cz, Pz]: 350–410 ms). For both components, separate ANOVAs were computed to test our hypotheses.

P3a (290–350 ms): In line with our first hypothesis, the deviant event (recipient non-intended) elicited a larger P3a component as compared to the standard event (recipient intended). Correspondingly, a significant effect of the factor *recipient* was found for the P3a (Table 2). Fig 1B suggests that an effect of intervention frequency can be found for the non-intended, but not for the intended outcome. In the former condition, P3a amplitude was more expressed if frequency of intervention was low. This observation is supported by the significant interaction of the factors *intervention frequency* and *recipient*. Post-hoc comparisons confirmed that an effect of intervention frequency can be observed for the non-intended outcome, *F*(1,50) = 98.17, *p* < .001, η_p_^*2*^ = .152, but not for the intended outcome, *F*(1,50) = .64, *p* = .427, η_p_^*2*^ = .013.

**Fig 3:**
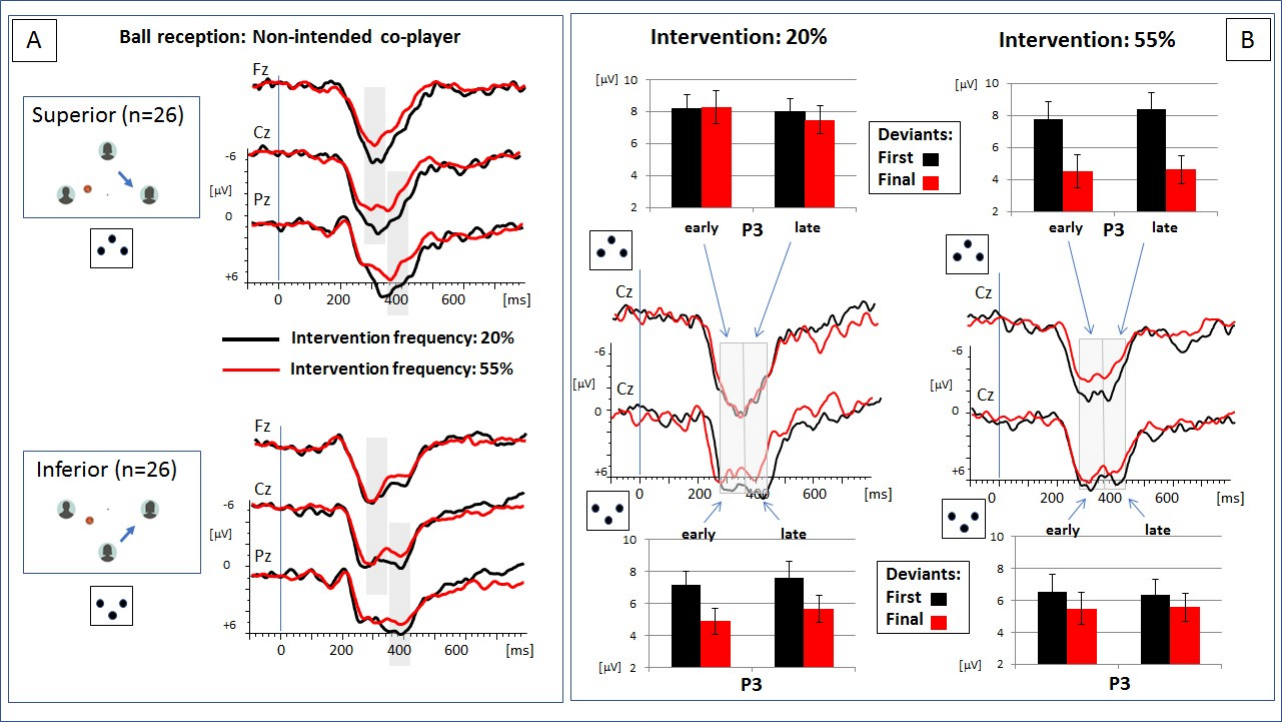
Effect of verticality on the processing of the deviant event (non-intended outcome): (A) ERP effect of intervention frequency separated for the vertical position of the participants’ avatar: At superior position, the P3 amplitude effect is clearly expressed for the fronto-central P3a and centro-parietal P3b amplitude. At inferior position, effects are reduced and restricted to the centro-parietal P3b amplitude. (B) ERP response at electrode Cz to the first and final deviants within an experimental block. Effects are separated for the intervention frequency (20%: left column vs. 55%: right column) and vertical position of the avatar (superior: upper row vs. inferior: lower row). If intervention frequency is low (20%), P3 responses to the final deviants is significantly reduced at an inferior position but remains stable at a superior position. If intervention frequency is high (55%), the response to the final deviants is markedly reduced–independently of the vertical position.

The three-way interaction of the factors *verticality*, *intervention frequency* and *recipient* (Table 2) was significant for the ANOVA and the ANCOVA. The effect triggered a post-hoc analysis, separated for the event ‘recipient intended’ and ‘recipient non-intended’. The processing of the standard event (intended outcome of a ball throw) was not influenced by the factor *verticality*: P3a amplitude was neither affected by the factor *verticality*, *F*(1,50) = .70, *p* = .407, η_p_^*2*^ = .014 (*F(1,49) = .46, *p* = .502, η_p_^*2*^ = .009), nor by the interaction of the factors *intervention frequency* and *verticality*, *F*(1,50) = 1.27, *p* = .265, η_p_^*2*^ = .025 (*F(1,49) = 2.12, *p* = .152, η_p_^*2*^ = .042). In contrast, the processing of the deviant event (non-intended outcome) was found to be influenced by the experimental factors. As shown in Fig 3A, P3a amplitude was more expressed in case of a low as compared to a high intervention frequency–only if the participants’ avatar was at a superior position. The ANOVA confirmed a significant interaction of the factors *intervention frequency* and *verticality*, *F*(1,50) = 4.41, *p* = .041, η_p_^*2*^ = .081 (*F(1,49) = 5.07, *p* = .029, η_p_^*2*^ = .094). In line with the visual impression (Fig 3A), P3a amplitude was more expressed in case of a low as compared to a high intervention frequency for the superior group, *F*(1,50) = 3.70, *p* = .060, η_p_^*2*^ = .069, but not for the inferior group, *F*(1,50) = 0.37, *p* = .546, η_p_^*2*^ = .012.

P3b (350–410 ms): The mean P3b amplitude was more expressed when the non-intended co-player received the ball (factor *recipient*, see Fig 1A). Moreover, the effects of probability of an intervention extended to the P3b amplitude: As for the early P3, the effect of intervention frequency appears to be restricted to the non-intended outcome (Table 2, *recipient* by *intervention frequency* interaction). Post-hoc comparisons triggered by the significant interaction confirmed that an effect of intervention frequency can be observed for the non-intended outcome, *F*(1,50) = 8.99, *p* = .004, η_p_^*2*^ = .152, but not for the intended outcome, *F*(1,50) = 0.19, *p* = .669, η_p_^*2*^ = .004. The factor *verticality* did not influence the intervention effect on the P3b amplitude systematically (see Table 2).

### ERP data: Adaptation effects within an experimental block

In line with previous ERP studies running an exclusionary cyberball [28, 42, 55], we hypothesized a decrease in P3 amplitudes within the experimental blocks. To account for the difference in intervention frequency between the two experimental blocks (20% vs. 55%), the analysis of adaptation effects focused on the averaged response to the first ten (first half) and to the final ten (second half) deviant events (*non-intended recipient)* within an experimental block (see methods). As shown above, the deviant event provoked a significant P3a and P3b response and was sensitive to the experimental factors. The changes in amplitude were analyzed separately for each experimental block. Results of the statistical analysis are provided in Tables 3 and 4.

**Table 3:** Descriptive statistics for the ERP data (intervention frequency 20%) for the non-intended outcome, separated for, assignment (vertical position: inferior vs. superior) and half (first and final deviants within a block). To each mean, the upper and lower limits of confidence intervals (95%) are additionally provided in brackets. ANOVA results for the factor vertical position are additionally controlled for the covariate 'age of participant' in an ANCOVA. All ANCOVA results are indexed by an asterisk (*).

Intervention Frequency	20%	
Vertical Position	Inferior	Superior
**P3a amplitude (290-350ms): first deviants**	7.13 μV (5.43 μV, 8.83 μV)	8.21 μV (6.51 μV, 9.91 μV)
**P3a amplitude (290-350ms): final deviant**	4.87 μV (3.23 μV, 6.52 μV)	8.27 μV (6.62 μV, 9.91 μV)
Effect (Half)	F(1,50) = 3.76, p = .058, η_p_^2^ = .070	
Effect (Position)	*F(1*,*50) = 4*.*67*, *p =* .*035*, *η*_*p*_^*2*^ *=* .*085 F(1*,*49) = 4*.*63*, *p =* .*036*, *η*_*p*_^*2*^ *=* .*086)*	
Effect (Half x Position)	*F(1*,*50) = 4*.*21*, *p =* .*045*,*η*_*p*_^*2*^ *=* .078 (*F(1,*49) = 4*.*70*, *p =* .*035*,*η*_*p*_^*2*^ *=* .*088)*	
**P3b amplitude (350-410ms): first deviants**	7.59 μV (5.50 μV, 9.68 μV)	8.02 μV (5.93 μV, 10.11 μV)
**P3b amplitude (350-410ms): final deviant**	5.36 μV (3.63 μV, 7.10 μV)	6.49 μV (4.76 μV, 8.22 μV)
Effect (Half)	F(1,50) = 2.78, p = .102, η_p_^2^ = .053	
Effect (Position)	F(1,50) = 1.04, p = .312, η_p_^2^ = .020 (*F(1,49) = 1.22, p = .275, η_p_^2^ = .024)	
Effect (Half x Position)	F(1,50) = 0.95, p = .336, η_p_^2^ = .019 (*F(1,49) = 2.03, p = .16, η_p_^2^ = .040)	

**Table 4:** Descriptive statistics for the ERP data (intervention frequency 55%) for the non-intended outcome, separated for, assignment (vertical position: inferior vs. superior) and half (first and final deviants within a block). To each mean, the upper and lower limits of confidence intervals (95%) are additionally provided in brackets. ANOVA results for the factor vertical position are additionally controlled for the covariate 'age of participant' in an ANCOVA. All ANCOVA results are indexed by an asterisk (*).

Intervention Frequency	50%	
Vertical Position	Inferior	Superior
**P3a amplitude (290-350ms): first deviants**	5.41 μV (3.99 μV, 6.82 μV)	6.66 μV (5.25 μV, 8.08 μV)
**P3a amplitude (290-350ms): final deviant**	5.33 μV (3.68 μV, 6.98 μV)	5.50 μV (3.95 μV, 7.25 μV)
Effect (Half)	*F(1*,*50) = 4*.*83*, *p =* .*033*, *η*_*p*_^*2*^ *=* .*088*	
Effect (Position)	F(1,50) = 0.05, p = .825, η_p_^2^ = .001 (*F(1,49) = 0.31, p = .578, η_p_^2^ = .006)	
Effect (Half x Position)	F(1,50) = 1.45, p = .234, η_p_^2^ = .028 (*F(1,49) = 0.51, p = .474, η_p_^2^ = .011)	
**P3b amplitude (350-410ms): first deviants**	5.63 μV (3.92 μV, 7.91 μV)	7.50 μV (5.79 μV, 9.22 μV)
**P3b amplitude (350-410ms): final deviant**	5.27 μV (3.73 μV, 6.82 μV)	5.17 μV (3.63 μV, 6.72 μV)
Effect (Half)	*F(1*,*50) = 7*.*03*, *p =* .*011*, *η*_*p*_^*2*^ *=* .*123*	
Effect (Position)	F(1,50) = 0.03, p = .861, η_p_^2^ = .001 (*F(1,49) = 0.35, p = .56, η_p_^2^ = .007)	
Effect (Half x Position)	F(1,50) = 3.10, p = .085, η_p_^2^ = .058 (*F(1,49) = 1.17, p = .285, η_p_^2^ = .023)	

Low intervention frequency (20%): As shown in Fig 3B, a reduction of the P3a and P3b amplitudes appeared to depend on the vertical position: An amplitude difference between the responses to the first and final deviants was clearly expressed for participants with an avatar at an inferior position, but not for participants with an avatar at a superior position. The statistical analysis (see Table 3) confirmed a significant interaction of the factors *verticality* and *half* only for the early P3a. This interaction effect was also significant when *age of participant* was controlled for as a covariate. For this component, post-hoc comparisons confirmed that a significant amplitude reduction from the first to the final deviants was significantly expressed in the ‘inferior’ group, *F*(1,50) = 7.67, *p* = .008, η_p_^2^ = .235, but not in the ‘superior’ group, *F*(1,50) = .007, *p* = .936, η_p_^2^ = .000.

High intervention frequency (55%): Fig 3B indicates a clear adaptation effect, expressed for both components (P3a and P3b) and both groups (inferior and superior position): Both, early and late P3 amplitudes, were significantly reduced when the response to the first and the final deviants was compared. Although the amplitude reduction appears to be more pronounced in the group of participants with an avatar at a superior position, the statistical analysis (see Table 4) did not confirm an interaction with group assignment (factor *verticality*).

Control condition: To control for the specificity of the adaptation effects in ERPs, we analyzed the corresponding signals recorded in *intended outcome* condition (no intervention). Amplitudes in the P3a and P3b time range were not significantly affected by the experimental variables (position, half). This held for the analysis of the first as well as for the second block.

## Discussion

This research set out to test whether the predictions of an expectancy violation approach can be extended to a situation in which the participant is included in the virtual ball game (inclusionary cyberball), but in which interventions by a “supervisor” affected the outcome of an intended ball throw. The analysis was focused on the P3 components in the ERP providing a marker of the participants’ expectancy state. Based on previous findings in exclusionary cyberball studies, we predicted that an intervention by a “supervisor” affecting personal control triggers a P3 effect [36]. The expression of this effect was predicted to depend on the predictability (frequency) of the intervention and the vertical position of the participants’ avatar [6]. Finally, the vertical position was hypothesized to also affect the adaptation to the recurring interventions within an experimental block [42].

In line with our first hypothesis, an intervention evokes an initial fronto-central ERP positivity that shows the spatial and temporal characteristics of a P3a complex [31, 44]. This indicates that the processing of an intervention shares the characteristics of a deviant event which affects the attentional allocation and involves the activation of a frontal attention network. Subsequently, the intervention also elicits a centro-parietal P3b component. Comparable to the processing of a target event in exclusionary cyberball (ball reception, see [36]), an intervention can be defined as a self-relevant event [56] which triggers mnestic processes (e.g. context updating process [30]). The early fronto-central (P3a) and the late centro-parietal (P3b) components differ with respect to topography and latency but are also differently affected by the experimental manipulation. Differences between the functional characteristics of the components will be discussed in the following.

Confirming the results of a previous exclusionary cyberball study [35], the expression of the P3b, but not of the P3a, is primarily affected by the *frequency of intervention*. This result is in line with the idea that the P3b amplitude is related to the manipulation of the likelihood of an event [45]. The change in likelihood of the deviant event, however, did not affect the processing of the complementary ‘standard’ event (intended recipient) for which P3b amplitude remained stable. This finding contrasts results from the exclusionary cyberball (ball reception ‘self’ vs. ‘others’, see [57]) and indicates that the underlying cognitive process of context updating [30] is exclusively triggered by the intervention of the supervisor challenging the participants’ control. We tentatively suppose that the result of the updating process serves the post-hoc estimation of the frequency of the supervisor’s activity. Testing this idea would be a fruitful avenue for future research.

The expression of the P3a component is less clearly affected by the *frequency of intervention*, and its activation apparently relies in a different probabilistic module [45]. However, the expression of the fronto-central P3a component was modulated by the *vertical* position of the participant’s avatar, thus supporting our second hypothesis. In case of low intervention frequency, the P3a response to the deviant event (non-intended recipient) was significantly enhanced in participants with an avatar at a superior position. A similar effect of verticality was also obtained in the exclusionary cyberball when a low frequency of ball reception signaled social exclusion [6, 42]. For both situations, social exclusion and loss of control, participants assigned to a superior position revealed an enhanced sensitivity to rare unexpected events.

Previous studies [6, 42] related the ERP effect of verticality in the processing of low-frequent aversive events to a differential self-assignment of social power [40]. The current ERP data suggest that this effect is not restricted to expected social participation, but extends to expected personal control [46]: In contrast to an inferior position, a superior position is associated with an elevated expectation of involvement *and* control [1] as indexed by an enhanced P3 amplitude. Consequently, the intervention is less expected and attentional allocation is more pronounced. Importantly, the effect of a superior position did not expand to the second block in which the intervention frequency was markedly enhanced. In contrast to the exclusionary cyberball data [6, 42], however, the effects of verticality are only weakly expressed for the self-reports in the case of intervention cyberball: Although the self-assigned personal power ratings appear to be selectively adjusted in participants assigned to a superior position, this effect is modulated by the age of the participant (see below). We therefore suggest physiological data are more sensitive to detect a verticality-power-link [40] as compared to retrospective self-reports–at least in case of an intervention cyberball based on long interaction sequences.

The aforementioned idea of a differential adjustment of expectancies is substantiated by the analysis of adaptation effect within an experimental block. In line with our third hypothesis, we observed systematic fluctuations of the P3 amplitudes within the experimental blocks which signal a re-adjustment in the participants’ state of expectancy [48]. In previous exclusionary cyberball studies, the adaptation was more expressed in participants assigned to an avatar at an inferior position [42]. In case of low intervention frequency (block 1), this effect was replicated for the P3a amplitude in the intervention cyberball. We therefore conclude that an inferior vertical position not only prepares for exclusionary events [6], but also for restrictions in personal control. The adaptation of the P3 amplitude (see Fig 2B) reveals that this preparation allows a rapid re-adjustment of expected control–independently of the frequency of deviant signals affecting personal control. Consequently, less attentional resources are directed to recurring interventions. In contrast, a superior position is associated with higher self-assigned personal control and participants are not prepared for the processing of interventions. Consequently, a re-adjustment of expectations is not triggered by occasional interventions (here: intervention frequency of 20%) and attentional allocation to a deviant is therefore not reduced. Importantly, this effect was restricted to the low-frequent intervention condition and did not expand to the high-frequent intervention condition.

This pattern of ERP results is congruent with specific theories from social psychology on power and control. Following the idea of a verticality-power-link [40], the physiological data confirm that self-assigned power can protect people psychologically *from* influence [58]. The differences in the attentional allocation depending of vertical position might also contribute to power-induced asymmetries obtained in the mirroring of social interaction partners in a previous study on motor resonance [59]. The differential adaptation effect within an experimental block (superior vs inferior vertical position) might signal that effects of self-assigned power crucially rely on the resistance to re-adjust expectations. This resistance, however, can be overruled if the frequency of intervention is increased. This indicates that recurring and *predictable* deviant events elicit a functional adaptation of expected control independently of self-assigned social power.

More importantly, this research is in line with the recent call to adopt overarching theoretical frameworks to explain associated psychological phenomena [60]. Both, the processing of social exclusion and the processing of loss of control, can be explained in terms of an expectancy violation process. In addition, the general inconsistency compensation model [17] also offers an explanation for the adaptation phenomena observed for the P3 component: According to the model, adaptation is not a mere passive process but reflects an active compensatory process. We suppose that the accommodative or affirmative behavior–following a loss of control [5] or social exclusion [12]–is accompanied by a re-adjustment of expected personal control or expected social participation, respectively. Evidence for this idea could be provided in further studies exploring whether the behavioral compensation process is differently expressed when the self-assignment of social power is high or low.

Our study is based on a virtual ball tossing game. It is notable that a recent ERP study using the Lunchroom task [61] also obtained a P3 effect in an socially aversive condition (exclusion), but that this effect was due to the recruitment of an early alarm system [29, 62]. However, the differential adaptation of the P3 amplitudes depending on vertical position–obtained in exclusion [42] and loss of control–appears to be more compatible with an expectancy account and defines a challenge for the hypothesized preattentive alarm system. Moreover, we assume that the modified cyberball paradigm introduced here can be expanded to research on the interplay between different social threats, and its effect on aggression [63]. Obviously, several boundary conditions of the effects reported here must be considered: First, differences in adaptability between participants (or groups) can only be detected in long interactions sequences, and if the aversive event is not highly predictable. Interestingly, the standard (exclusionary) cyberball setup does not meet these requirements so that these differences are probably masked in standard social exclusion cyberball setups. Second, constraints on generality related to the sample of participants must be considered: Despite of the robustness of the–related–exclusionary cyberball effect [24] and the verticality-power relationship [40, 64], we cannot exclude a bias in the belief in personal control in the undergraduate students examined. The response to intervention might be moderated by the educational level or by age. Please note that the experimental groups differed with respect to mean age (‘superior‘: 25.96 vs ‘inferior‘: 22.50 years). The results of the ANCOVA signaled that the factor age of participant did not affect the ERP data (see Tables 2 and 3), but moderated the statistical effects for the self-report (see Table 1: personal power). Further research using questionnaire data therefore has to consider that an effect of age on the self-assignment of personal power might be present even within a limited age range. Third, the order of experimental conditions was not counter-balanced and, therefore, contrast effects (low-to-high vs high-to-low intervention frequency) already reported in the exclusionary cyberball [35] cannot be considered. Given the marked adaptation effects observed in the high frequency condition, a corresponding contrast effect is highly likely in the intervention cyberball, as well, and should be examined in further studies. Fourth, we have to consider that the split-half ERP analysis is based on a low number of trials. Unfortunately, the analysis of the covert adaptation process necessarily relies on the analysis on a restricted number of critical events (here: 7–10). Nevertheless, the P3 signal was reliable (see above), and the differential adaptation effect induced by verticality has also been observed in social exclusion [7]. Finally, our ERP analysis is only based on midline electrodes. A more fine-graded analysis of the spatial distribution P3 effect will require an increase of active leads in future studies. We have no reason to believe that the results depend on other characteristics of the participants, materials, or context.

In conclusion, this research supports the notion that a *loss of control* in a modified version of the cyberball paradigm defines an aversive event which violates subjective expectation. Consistent with previous ERP results on *social exclusion* using a standard cyberball paradigm, the degree of expectancy violation is influenced by the predictability of the aversive events and the vertical position assigned: A superior position prevents the adaptation to occasional aversive events in longer interaction sequences. The electrophysiological effects can be related to a differential self-assignment of power depending on verticality. Overall therefore, this research supports the notion of a common cognitive mechanism in reactions to social exclusion and loss of control based on an inconsistency in expectancy states.

## Supporting information

S1 Appendix Results of additional analyses. S1 Appendix provides the analysis of the ERP effects triggered by the ball reception of the participant (Figs A and B, Tables A to C): Effects of intervention frequency were analyzed separately for the experimental groups (superior and inferior position). Moreover, the results of the experimental effects on the four NTQ scales (belonging, self-esteem, control, and meaningful existence) are provided (Tables D and E).
(PDF)
