# Supplementary material for: Loss of control as a violation of expectations: Testing the predictions of a common inconsistency compensation approach in an inclusionary cyberball game
Source: PLoS One. 2019 Sep 9;14(9):e0221817. doi: 10.1371/journal.pone.0221817 (PMC6733461; doi:10.1371/journal.pone.0221817)
Supplement: S1 Appendix — provides the analysis of the ERP effects triggered by the ball reception of the participant (Figs A and B, Tables A to C): Effects of intervention frequency were analyzed separately for the experimental groups (superior and inferior position). Moreover, the results of the experimental effects on the four NTQ scales (belonging, self-esteem, control, and meaningful existence) are provided (Tables D and E). (PDF) [file pone.0221817.s001.pdf]

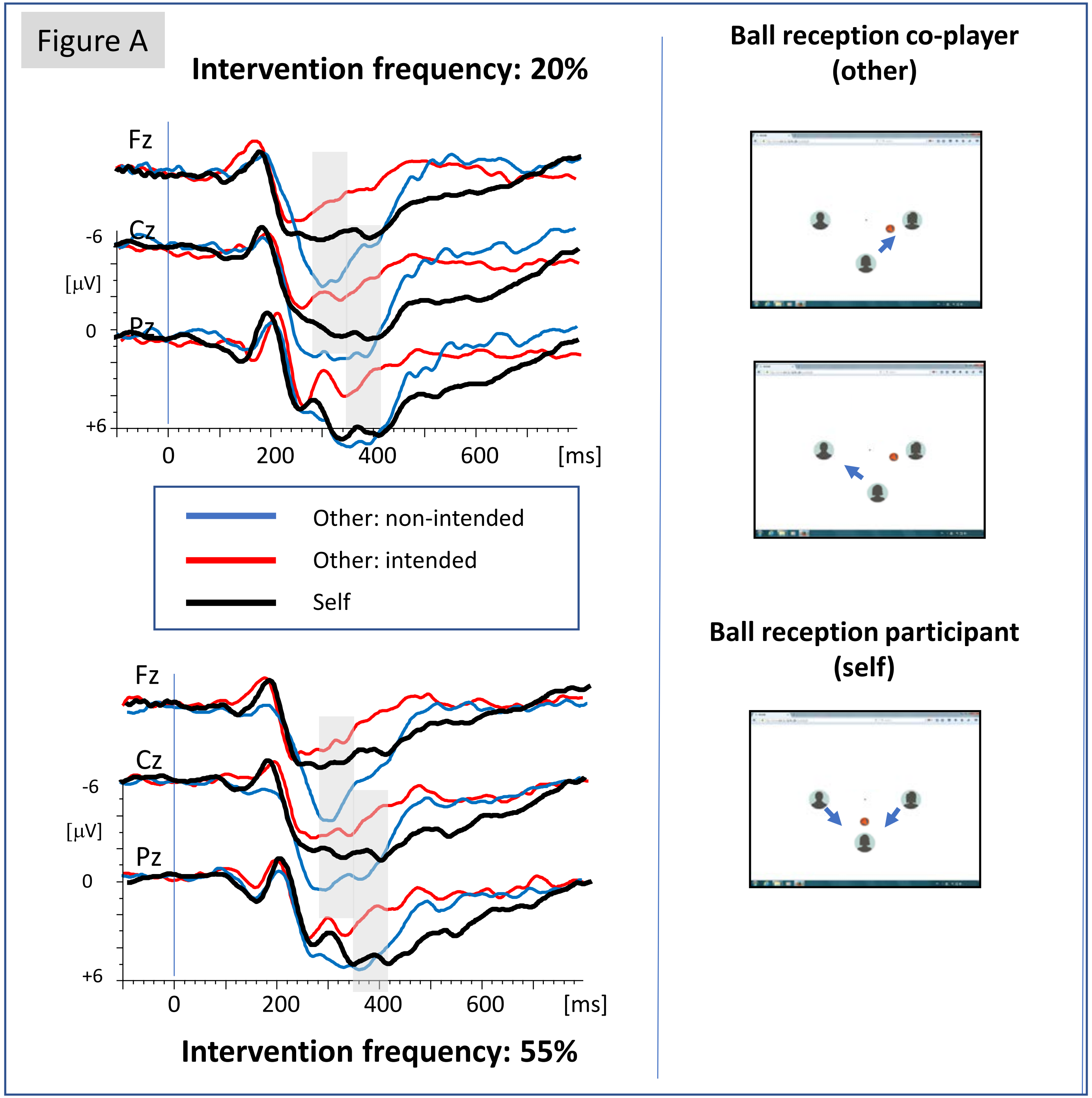

Table A

P3 amplitudes (self)

Early P3 (290-350 ms)

Intervention Frequency	Mean	CI 95% (lower)	CI 95% (upper)
20%	4.31	3.52	5.11
55%	3.71	2.93	4.49

P3 amplitudes (self)

Late P3 (350-410 ms)

Intervention Frequency	Mean	CI 95% (lower)	CI 95% (upper)
20%	5.65	4.68	6.63
55%	4.78	3.98	5.57

Figure B

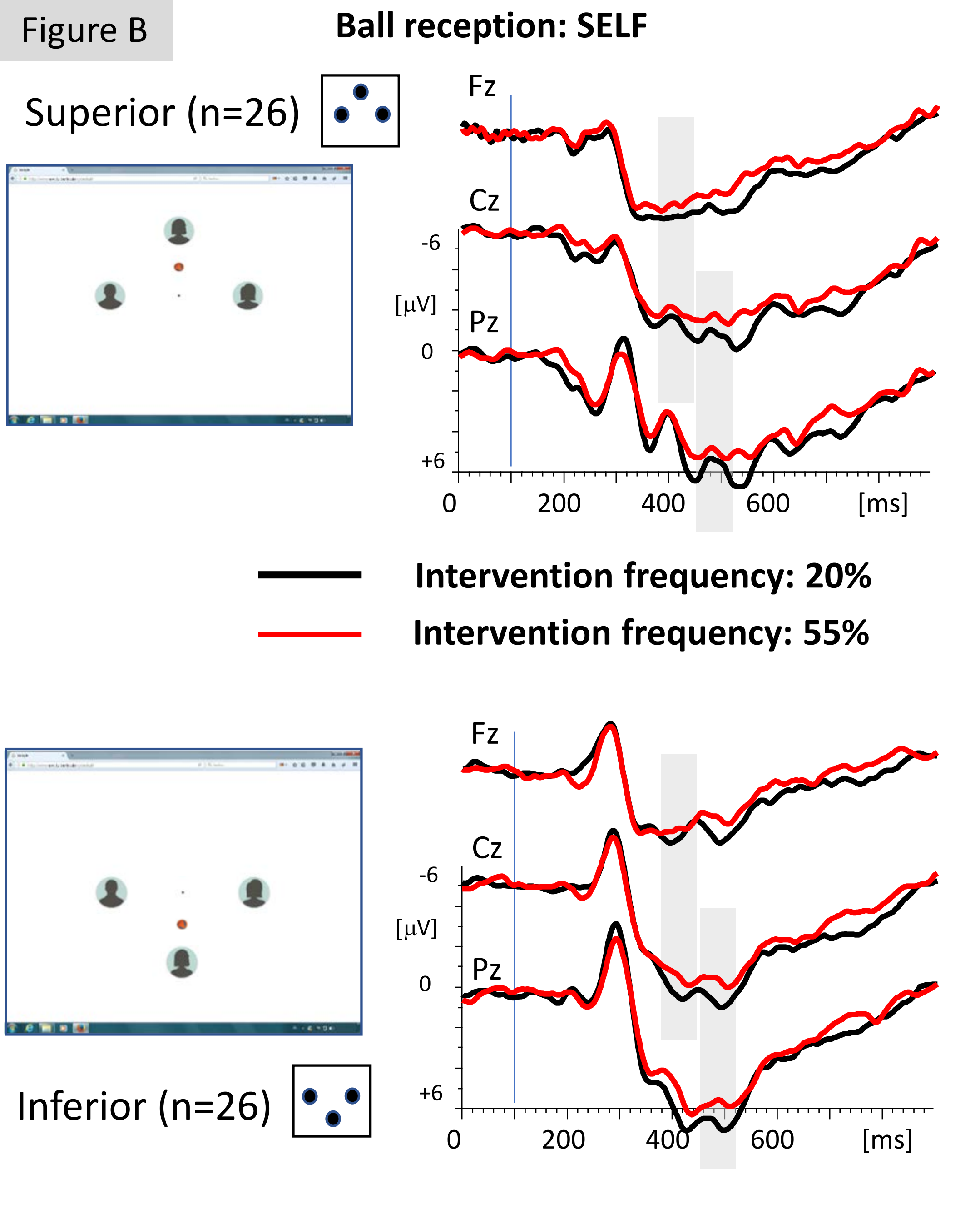

Table B

Vertical Position: Superior

Early P3 (290-350 ms)

Intervention Frequency	Mean	CI 95% (lower)	CI 95% (upper)
20%	4.38	3.27	5.50
55%	3.65	2.55	4.74

Late P3 (350-410 ms)

Intervention Frequency	Mean	CI 95% (lower)	CI 95% (upper)
20%	5.24	3.87	6.61
55%	4.46	3.35	5.57

Vertical Position: Inferior

Early P3 (290-350 ms)

Intervention Frequency	Mean	CI 95% (lower)	CI 95% (upper)
20%	4.24	3.11	5.38
55%	3.77	2.66	4.89

Late P3 (350-410 ms)

Intervention Frequency	Mean	CI 95% (lower)	CI 95% (upper)
20%	6.06	4.67	7.45
55%	5.09	3.98	6.23

Table C

Statistical Analysis: Early P3 (self)

IF = Intervention Frequency
VP = Vertical Position

Factor IF : $F(1,50)=3.06$, $p=.087$, $\eta_p^2=.058$

Factor VP : $F(1,50)=0.01$, $p=.924$, $\eta_p^2=.000$

Interaction VP x IF : $F(1,50)=0.08$, $p=.777$, $\eta_p^2=.002$

Statistical Analysis: Early P3 (self)

IF = Intervention Frequency
VP = Vertical Position

Factor IF : $F(1,50)=5.93$, $p=.019$, $\eta_p^2=.106$

Factor VP : $F(1,50)=0.86$, $p=.357$, $\eta_p^2=.017$

Interaction VP x IF : $F(1,50)=0.09$, $p=.768$, $\eta_p^2=.002$

Table D

NTQ: Belonging

Intervention Frequency	Position	Mean (lower)	CI 95% (upper)	CI 95%
20%	superior	3.77	3.42	4.11
55%	superior	3.85	3.52	4.17
20%	inferior	3.83	3.49	4.18
55%	inferior	3.78	3.46	4.11

Statistical Analysis:

IF = Intervention Frequency
VP = Vertical Position

Factor IF : $F(1,50)=0.01$, $p=.913$, $\eta_p^2=.000$

Factor VP : $F(1,50)=0.00$, $p=1.000$, $\eta_p^2=.000$

Interaction VP x IF : $F(1,50)=0.30$, $p=.585$, $\eta_p^2=.006$

NTQ: Self Esteem

Intervention Frequency	Position	Mean (lower)	CI 95% (upper)	CI 95%
20%	superior	3.53	3.23	3.83
55%	superior	3.53	3.20	3.85
20%	inferior	3.40	3.10	3.69
55%	inferior	3.30	2.97	3.62

Statistical Analysis:

IF = Intervention Frequency
VP = Vertical Position

Factor IF : $F(1,50)=0.20$, $p=.656$, $\eta_p^2=.004$

Factor VP : $F(1,50)=0.91$, $p=0.344$, $\eta_p^2=.018$

Interaction VP x IF : $F(1,50)=0.20$, $p=.656$, $\eta_p^2=.004$

Table E

NTQ: Meaningful Existence

Intervention Frequency	Position	Mean (lower)	CI 95% (upper)	CI 95%
20%	superior	4.10	3782	4.42
55%	superior	4.18	3.83	4.53
20%	inferior	3.30	3.97	4.61
55%	inferior	4.23	3.88	4.57

Statistical Analysis:

IF = Intervention Frequency
VP = Vertical Position

Factor IF : $F(1,50)=0.04$, $p=.951$, $\eta_p^2=.000$

Factor VP : $F(1,50)=0.34$, $p=0.564$, $\eta_p^2=.007$

Interaction VP x IF : $F(1,50)=0.46$, $p=.502$, $\eta_p^2=.009$

NTQ: Control

Intervention Frequency	Position	Mean (lower)	CI 95% (upper)	CI 95%
20%	superior	2.28	1.97	2.59
55%	superior	2.04	1.76	2.32
20%	inferior	2.35	2.04	2.66
55%	inferior	2.09	1.81	2.37

Statistical Analysis:

IF = Intervention Frequency
VP = Vertical Position

Factor IF : $F(1,50)=6.09$, $p=.017$, $\eta_p^2=.108$

Factor VP : $F(1,50)=0.09$, $p=0.763$, $\eta_p^2=.002$

Interaction VP x IF : $F(1,50)=0.08$, $p=.930$, $\eta_p^2=.000$
